# Effects of Titanium Dioxide Nanoparticles on Red Clover and Its Rhizobial Symbiont

**DOI:** 10.1371/journal.pone.0155111

**Published:** 2016-05-12

**Authors:** Janine Moll, Annette Okupnik, Alexander Gogos, Katja Knauer, Thomas D. Bucheli, Marcel G. A. van der Heijden, Franco Widmer

**Affiliations:** 1 Institute for Sustainability Sciences ISS, Agroscope, Zurich, Switzerland; 2 Plant-Microbe-Interactions, Department of Biology, Utrecht University, Utrecht, the Netherlands; 3 Federal Office for Agriculture FOAG, Berne, Switzerland; 4 Institute of Evolutionary Biology and Environmental Studies, University of Zurich, Zurich, Switzerland; Institute for Materials Science, GERMANY

## Abstract

Titanium dioxide nanoparticles (TiO_2_ NPs) are in consideration to be used in plant protection products. Before these products can be placed on the market, ecotoxicological tests have to be performed. In this study, the nitrogen fixing bacterium *Rhizobium trifolii* and red clover were exposed to two TiO_2_ NPs, i.e., P25, E171 and a non-nanomaterial TiO_2_. Growth of both organisms individually and their symbiotic root nodulation were investigated in liquid and hydroponic systems. While 23 and 18 mg l^-1^ of E171 and non-nanomaterial TiO_2_ decreased the growth rate of *R*. *trifolii* by 43 and 23% respectively, P25 did not cause effects. Shoot length of red clover decreased between 41 and 62% for all tested TiO_2_ NPs. In 21% of the TiO_2_ NP treated plants, no nodules were found. At high concentrations certain TiO_2_ NPs impaired *R*. *trifolii* as well as red clover growth and their symbiosis in the hydroponic systems.

## Introduction

Titanium dioxide nanoparticles (TiO_2_ NPs) are manufactured worldwide at an estimated quantity up to 88’000 t y^-1^, making them one of the most used NPs [1]. TiO_2_ NPs are used for instance in cosmetics, plastics and paint [2–4]. Also in food TiO_2_ particles are used for white coloring and are labeled in Europe as E171 independent of a certain particle size [5]. These applications of TiO_2_ NPs have resulted in considerable releases into the environment. Due to the larger quantities applied food grade TiO_2_ NP pigments (e.g. E171) have a higher probability to enter the environment than photocatalysts (e.g., P25) [6–7]. In Europe it has been estimated that TiO_2_ NP inputs into soils may reach 0.13 μg kg^-1^ y^-1^ and, if sewage sludge is applied, may be as high as 1200 μg kg^-1^ y^-1^ [8]. Because of their photo-protective and photocatalytic properties, TiO_2_ NPs are also considered for use in plant protection formulations to modify the lifetime of active ingredients [9–10]. Future application of plant protection formulations could result in estimated additional TiO_2_ NP input into soils ranging from 3 to more than 5000 μg kg^-1^ y^-1^ [9–11]. Therefore, it is important to determine possible effects of TiO_2_ NPs on plants, soil organisms and ecosystem functions as basis for an environmental risk assessment.

Legumes and their nitrogen fixing bacterial symbionts are important providers of nitrogen in agricultural systems, representing a central ecosystem service [12]. To perform nitrogen-fixation a complex sequence of signaling between rhizobia and plants takes place, which results in morphological alterations of root hairs and nodule formation [13]. An important legume is red clover (*Trifolium pratense*), which is used as fodder crop and green manure due to its symbiosis with the nitrogen fixing bacterium *Rhizobium trifolii*. Up to 373 kg nitrogen ha^-1^ y^-1^ can be fixed by the symbionts *R*. *trifolii* and *T*. *pratense* [14]. The importance of legumes for agricultural systems is expected to increase in the future because legumes increase nitrogen availability in soil and reduce the reliance on mineral nitrogen (N) fertilization [14]. Effects of TiO_2_ NPs on nitrogen-fixation have been reported for other legume-rhizobia models such as pea [15] and barrel clover [16]. For these reasons, it is important to investigate whether TiO_2_ NPs have adverse effects also on other symbiotic legume-rhizobia interactions such as e.g., red clover and *R*. *trifolii*

Hydroponic systems are suitable to assess plant development under highly controlled conditions. In particular, exposure to NPs can more easily be controlled and effective NP concentrations and particle size can be determined over time, which is of importance when assessing effects on plant performance [17]. However, many NPs tend to aggregate and sediment in growth media depending on, e.g., NP concentration, pH, ionic strength, humic acid and protein content of the medium [18–19]. Therefore it is important to determine the actual exposure concentration and the NP quality during exposure [20]. Various studies have reported experiments with TiO_2_ NPs in hydroponic systems, which have revealed contrasting effects on plant growth and biomass production and nitrogen fixation [15, 21–24]. These effects may depend on plant species as well as NP types, concentrations, and qualities. To the best of our knowledge, effects of TiO_2_ NPs on the important fodder crop red clover and its symbiosis with *R*. *trifolii* have not been assessed yet.

In this study, we used a liquid culture system to assess growth of *R*. *trifolii* exposed to different TiO_2_ NPs. We then developed a small scale hydroponic system to assess the impact of TiO_2_ NPs on red clover and root nodulation by *R*. *trifolii*. E171 (100% anantase) was chosen because food grade TiO_2_ NPs have the highest probability to get released to the environment [6–7]. As a non-nano material [25] we chose an anatase particle with average particle size larger than 100 nm (non-nanomaterlal (NNM) TiO_2_). To assess whether a fraction of rutile crystal structure changes potential effects, we also included P25 (20% rutile, 80% anatase) in our assessment. TiO_2_ NPs can be dissolved at low pH (pH<3). However, at pH between 3 and 8 no ions were detected as shown for P25 [26–27]. We chose ZnSO_4_ as a positive control because it has been reported to affect plant growth [28–29]. We aimed to determine whether (1) TiO_2_ NP concentrations and qualities changed over the duration of the experiment, and whether (2) growth rate of *R*. *trifolii*, (3) growth of red clover, and/or (4) nodule formation by *R*. *trifolii* on clover roots are affected.

## Material and Methods

### Nanoparticles

TiO_2_ NPs were P25 (80% anatase, 20% rutile, Sigma-Aldrich, USA, Art. No. 718467) and Hombitan FG, which we refer to as E171 (100% anatase, Sachtleben Pigments, Germany). Additionally, a NNM TiO_2_ preparation (100% anatase, Sigma Aldrich, Art. No. 232033) was chosen as non-nano material [25] containing less than 50% NPs (size distribution). All of these TiO_2_ NPs and the NNM TiO_2_ were uncoated. As a positive control, ZnSO_4_^.^7H_2_O (Sigma-Aldrich) was used. Size distributions of primary TiO_2_ NPs were measured by transmission electron microscopy (TEM). For this, TiO_2_ NPs, i.e., P25, E171 and NNM TiO_2_, were suspended in MQ water (Milli-Q Gradient A10, Millipore Corporation, Molsheim, France) by sonication in an ultrasonic bath (Sonorex digital 10 P, Bandelin, Germany) for 30 min at 720 W. A drop of the resulting suspension was then air-dried on a formvar/carbon coated TEM grid (Plano, Wetzlar, Germany) and visualized using a Tecnai G2 Spirit transmission electron microscope (FEI, Delmont, PA, USA). Electron micrographs were analyzed with ImageJ (S1 Appendix) [30]. P25 particles were the smallest particles with an average diameter of 29±9 nm (n = 92) confirming the manufacturer’s specification of 21 nm. The size of E171 and NNM TiO_2_ were on average 92±31 nm (n = 52) and 145±46 nm (n = 49), respectively. NPs, i.e., particles with at least one dimension below 100 nm, were 100% for P25, and 69% for E171. NNM TiO_2_ contained 20% NPs and thus is referred to a non-nano material [25]. No larger particle sizes for the NNM TiO_2_ control were chosen, because suspended particles needed to be stable over time for the exposure experiments. Using E171 and NNM TiO_2_ allowed us to compare a nano-material with a non-nano-material.

### Preparation of NPs

Because the growth media used needed to be sterile, surface sterilization of the NPs was performed. TiO_2_ NPs (5 mg and 2.5 mg for liquid cultures and hydroponic system, respectively) were sterilized in 70% ethanol (0.4 ml) for 1h at 60°C. TiO_2_ NPs in ethanol were transferred antiseptically with a pipette to Schott bottles containing 100 ml yeast mannitol broth (YMB) for *R*. *trifolii* liquid cultures or Fåhraeus medium (FM) for hydroponic cultures [31]. For controls without NPs, 0.4 ml 70% ethanol were added. Natural organic matter (40 mg l^-1^, NOM, IHSS Suwannee River, RO isolation 2R101N, USA) was added to both media. The amount of NOM suitable for stabilization of the suspensions in our systems was tested using a concentration series of NOM in advance of the presented experiments. For better initiation of plant growth and assessment of nitrogen uptake in plants, KNO_3_ (0.001 M, 4% ^15^N, Cambridge Isotope Laboratories, USA) was added to the FM. Media were sonicated for 1 h at 720 W. The suspensions for the *R*. *trifolii* liquid cultures were sedimented for 24 h, and 50 ml of the supernatant was diluted to the final concentrations (1:0, 1:3, 1:9 and 1:27) and used for exposure experiments. For the hydroponic cultures, the NP containing medium (FM) was directly diluted (1:0 and 1:1 with FM) and used after sonication. The actual concentration of the undiluted NP suspensions was determined as total titanium from 3 ml of the suspensions (n = 3) by ammonium persulfate digestion as described by Khosravi et al. [32].

### Rhizobium trifolii

The nitrogen fixing bacterium *R*. *trifolii* 30141 (DMSZ, Germany; NCBI Gen Bank AY509900.1) was used for the experiments. We selected for rifampicin resistance on yeast mannitol agar (YMA, [31]) by sequential plating on increasing concentrations of rifampicin up to 250 μg ml^-1^ [33]. Resistant *R*. *trifolii* were grown in yeast mannitol broth (YMB) at 26°C and 150 rpm for 5 d [31]. *R*. *trifolii* were stored in 15% glycerol at -70°C until use.

### Exposure of *R*. *trifolii* in liquid cultures

Effects of TiO_2_ NPs on *R*. *trifolii* growth rate in YMB were assessed similar as in the study of Bandyopadhyay et al. [34] by measuring optical density (OD) at 620 nm using a spectrophotometer (Infinite F200, TECAN, Maennedorf, Switzerland). Controls without *R*. *trifolii* inoculation but the same NP concentrations as the treatments with *R*. *trifolii* were used for background OD determination. Background OD was subtracted from the OD of the samples with *R*. *trifolii* inoculation. Temperature was set to 26°C for optimal growth of *R*. *trifolii* (150 rpm, dark conditions, n = 4). *R*. *trifolii* was exposed for 32 h and subsamples for OD measurements were taken at t = 0 and from 26 h on every second hour. From each exponential part of the growth curve (S1 Appendix) a linear regression of ln-transformed OD over time was applied for determination of the growth rate.

### Red clover

Red clover (*Trifolium pratense* var. Merula) was used for the hydroponic experiments. Seeds were surface sterilized (10 min in 3% bleach and 5 min in 70% ethanol) and put into a hydroponic system adapted from Tocquin et al. [35]. Seeds were germinated in 200 μl pipet tips from which the front part was removed, and which were filled with 0.65% agar and 100 μg ml^-1^ rifampicin in an autoclaved, water filled pipet tip box in a growth chamber for 7 d (day: 16 h at 20°C and 250 μmol m^-2^ s^-2^ light, night: 8 h at 15°C, humidity 95%). Seedlings of similar height and root length were selected for the hydroponic experiment.

### Exposure in the hydroponic system

Effects of TiO_2_ NPs on red clover and symbiosis with *R*. *trifolii* were assessed in a hydroponic system (n = 6) consisting of test tubes (16 mm x 150 mm) containing 20 ml of the TiO_2_ NP suspensions in FM. All of the used TiO_2_ NP concentrations caused turbidity of the medium (S1 Appendix). Treatments with *R*. *trifolii* were inoculated with 1 ml of an overnight culture in YMB (2x10^7^ cells ml^-1^). Seedlings of red clover were transferred to the hydroponic system, and fixed with cotton. A cannula was inserted to allow addition of water and air with a syringe. Tubes were wrapped in aluminum foil to exclude light and hydroponic cultures were placed in a growth chamber for 28 days (16 h 20°C and 250 μmol m^-2^ s^-1^ light day and 8 h at 15°C night, humidity 95%). The medium was not mixed during exposure but was replaced weekly. Plants were watered with autoclaved water when the water level dropped below the end of the pipet tip. At harvest, roots were rinsed with deionized water and separated from the shoot. Main shoot and root length were measured, and the number of secondary roots, root tips, and nodules were counted. For determination of dry weight, shoots and roots were dried at 70°C until weight constancy. Shoots were ground in a ball mill (MM301, Retsch, Haan, Germany), and 2 mg shoot powder per sample were used for determination of ^14^N and ^15^N content (Isotope Ratio Mass Spectroscopy, Stable Isotope Facility of the University of Saskatchewan, Canada) as described by Arcand et al. [36]. In a further experiment, randomly selected nodules of six controls and six E171 treated red clover plants with and without inoculation of *R*. *trifolii* were surface sterilized and crushed on YMB agar [31]. If colonies were formed, they were plated on YMB agar containing 150 μg ml^-1^ rifampicin.

### Actual NP quality and concentrations in growth media

To verify whether the added quantities of TiO_2_ corresponded to the calculated TiO_2_ NP concentration, we measured the actual exposure of TiO_2_ NPs in the growth media, both for liquid cultures with *R*. *trifolii* growth and the hydroponic culture experiment. Total elemental titanium was determined in three ml suspension. Ammonium persulfate digestion [32] was used and concentration was determined with inductively coupled plasma optical emission spectroscopy [32] (ICP-OES: Spectro Arcos, Spectro, Germany). For the hydroponic system, this was repeated at every medium change, and for the *R*. *trifolii* liquid culture experiment, where the suspension was continuously mixed, the concentration was measured at the beginning of the experiment. Particle size and zeta potential (dynamic light scattering, DLS, Zetasizer Nano, Malvern Instruments, Germany) of the stock suspensions were determined at every medium change for the hydroponic experiment and at the beginning and end of the *R*. *trifolii* growth experiment to monitor agglomeration of NPs. Stability of the concentration of suspended TiO_2_ NPs in the hydroponic system, was determined after 18, 24, 42, 114 and 162 h for the top part (17 ml), where the roots were growing, and the bottom part (3 ml).

Coverage of roots with TiO_2_ NPs was estimated by analyzing scanning electron microscopy images (SEM) by applying a 3 μm raster and measuring the area of the TiO_2_ NPs within each square (Adobe Photoshop CS4 Extended 11.02). In total, 1117 squares on 10 different SEM images of different E171 treated root sectors and 1324 squares of control roots were analyzed. Sample preparation for SEM is explained in S1 Appendix.

### Statistics

All statistical analyses were performed with R [37]. For comparing the growth rates of *R*. *trifolii* liquid cultures, and the plant growth variables in the hydroponic system, a generalized linear model [38] was applied. P-values were adjusted for multiple testing according to Benjamini and Hochberg [39]. For *R*. *trifolii* liquid cultures each particle was tested in a separate experiment. Therefore relative growth rates were calculated to be able to compare these experiments. If the model assumptions for using a generalized linear model were not fulfilled (not normally distributed residuals (shapiro.test) and inhomogeneous variances (bartlett.test)), a Kruskal test (kruskal.test) followed by a Mann-Whitney test (wilcox.test) was conducted. For presence and absence data (e.g., nodules), a test of equal proportions (prop.test) was applied.

## Results

### Characteristics of TiO_2_ NPs in growth media

For assessing the agglomeration of TiO_2_ NPs particle size and zeta potential were determined. In YMB the average hydrodynamic diameters of TiO_2_ NPs determined with DLS were between 341 and 806 nm. Zeta potentials ranged between -29 and -33 mV (n = 3, Table 1). Initial titanium concentrations in YMB stock suspensions ranged from 18 to 24 mg l^-1^ (n = 3, Table 1, S1 Dataset).

**Table 1:** Analytical data of the TiO_2_ NPs suspended in YMB medium. Suspensions for the *R*. *trifolii* exposure experiment were assessed at the start of the experiment (t = 0) and at the end, i.e., after 34 h (n = 3).

	initial t = 0			after exposure t = 34 h	
Treatment	size [nm]	zeta potential [mV]	concentration [mg l^-1^]	size [nm]	zeta potential [mV]
P25	806±17	-29±1	23±5	879±174	-28±1
E171	341±3	-31±1	24±2	341±6	-32±2
NNM TiO_2_	356±1	-33±1	18±1	346±4	-33±2

In FM average hydrodynamic diameters were between 383 and 1077 nm (n = 3, Table 2, S2 Dataset). Zeta potential was between -21 and -30 mV (Table 2). The initial titanium concentrations of the stock suspensions in FM, ranged between 11 and 27 mg l^-1^ over four weeks (Table 2). While concentrations and zeta potentials revealed a moderate correlation of r = -0.48 (p = 0.013), and particle size and zeta potential of r = 0.55 (p<0.001), concentration and size of the NPs were not correlated r = -0.09 (p = 0.647). A decrease in the starting concentrations was observed in weeks 3 and 4.

**Table 2:** Analytical data of the TiO_2_ NP suspensions in Fåhraeus medium (FM). Suspensions of the hydroponic experiment were explored over four weeks (n = 3).

Particle	exposure week	conc.^1^ [mg l^-1^]	average size DLS^2^ [nm]	PDI	zeta-potential^2^ [mV]
P25	Week 1	27±2	876±46	0.49	-26±0
	Week 2	21±1	1663±248	0.85	-26±0
	Week 3	11	961±182	0.74	-24±0
	Week 4	12±0	1077±98	0.64	-21±1
E171	Week 1	21±4	383±10	0.24	-28±1
	Week 2	23±1	477±14	0.28	-28±1
	Week 3	16±0	524±19	0.39	-29±1
	Week 4	17±3	392±5	0.25	-25±1
NNM TiO_2_	Week 1	25±1	394±8	0.25	-30±0
	Week 2	17	446±16	0.29	-28±1
	Week 3	18±1	406±7	0.31	-27±0
	Week 4	17±1	467±21	0.33	-28±1

^1^Concentration of total titanium in the suspension at the beginning of the exposure week (n = 3) for the highest concentrations of each particle (2). The lower concentration (1) was diluted 1:1 with FM medium. For weeks two and three, two P25 and two NNM TiO_2_ samples were lost during digestion and thus only one sample could be used for determination of the concentration

^2^Stock suspension was measured at every medium change

**Fig 1:**
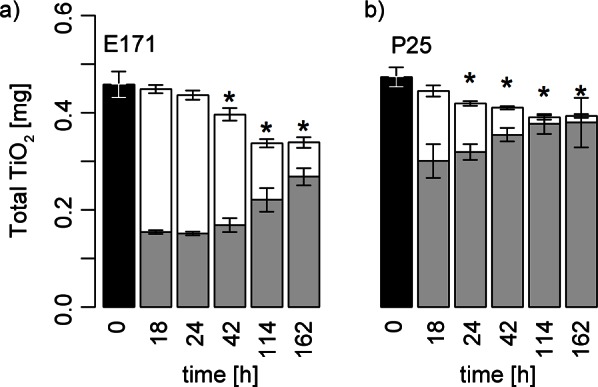
Total TiO_2_ suspended or sedimented in the hydroponic system. Red clover was exposed (n = 3) over 162 h to the two nanoparticles P25 and E171. TiO_2_ amounts of the pooled stock suspension is shown at t = 0 in black. TiO_2_ amounts of the top (white, 17 ml, in contact with roots) and bottom part (grey, 3 ml, including precipitate) are shown. Differences of the total TiO_2_ NP amount (bottom and top part together) to the total Ti amount at t = 0 are indicated with asterisks (p<0.05). Error bars indicate standard deviations (n = 3).

**Fig 2:**
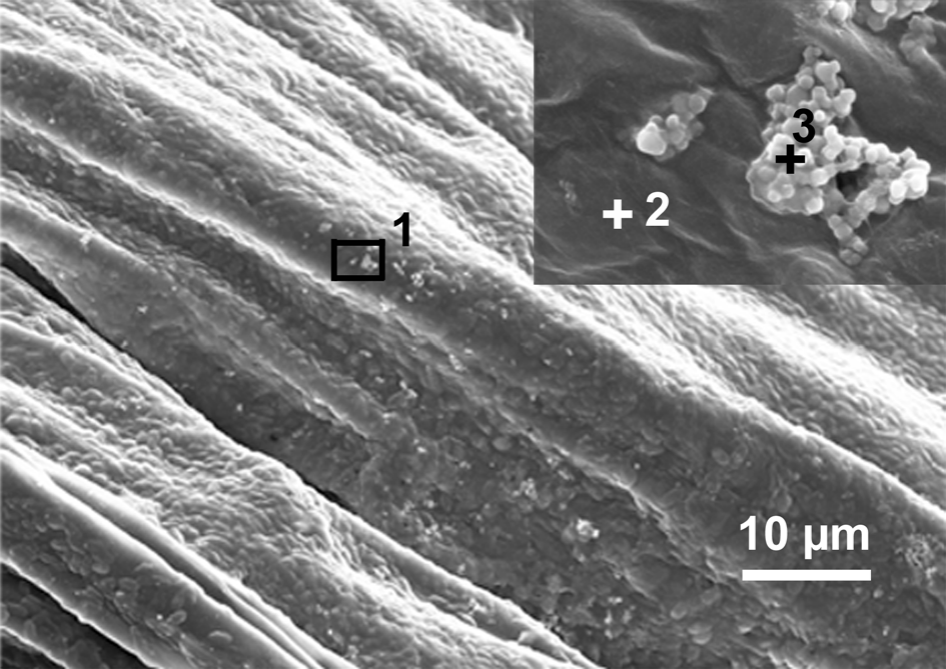
Scanning electron microscopy image of dried red clover root. Root surface from a 24 mg l^-1^ E171 treated plant is shown. The insert shows a magnification of 1, and from the spots 2 and 3 (+) X-ray fluorescence spectra were prepared revealing that spot 2 did not contain titanium while spot 3 contained titanium.

Sedimentation of the two nanoparticles, P25 and E171, in FM was determined over a 7 day period to monitor how exposure was changing over time. The total amount of titanium in the top part (17 ml) in contact with the red clover roots decreased by 85% for E171 and 98% for P25, when compared to the initial titanium concentration (Fig 1, S1 Appendix, S3 Dataset). In the bottom part (3 ml) at the end of the 7 d exposure, 59% of the initial amount of titanium was detected for E171 and 80% for P25. P25 sedimented faster than E171 compared to the respective control, which is consistent with the observation of the different particle sizes and zeta potentials (Table 2). Thus, compared to the initial titanium amounts in both treatments, 26% of E171 and 18% of P25 were not detectable and were most likely attached on the root surface (Fig 2) or the glass tube. The analysis of SEM images of rinsed root samples showed that on average 0.01±0.005 μm^2^ E171 covered 1 μm^2^ root surface. Additionally, TiO_2_ NPs formed a layer of white precipitate on the glass tube. However, its titanium content could not be quantified.

### Effects on *R*. *trifolii* in liquid cultures

**Fig 3:**
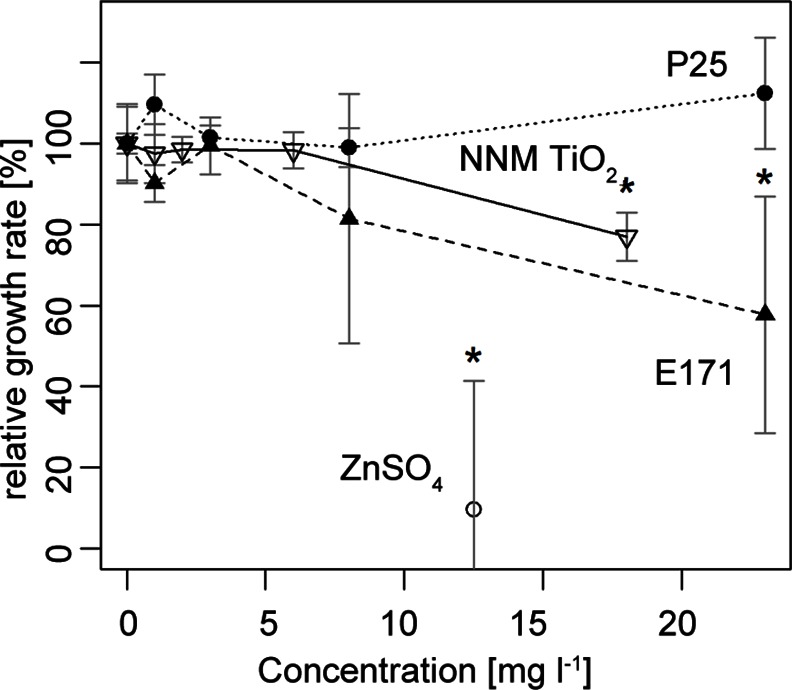
Relative growth rates of *R*. *trifolii* in YMB medium over a 32 h exposure assessed by optical density. *R*. *trifolii* growth rates were assessed in medium containing different actual concentrations and qualities of TiO_2_ NPs (P25 filled diamond, E171 filled triangle, NNM TiO_2_ inverted triangle, ZnSO_4_*7H_2_O circle) during the 34 h (n = 4). Stars indicate significant (p<0.05) differences from the control. Exponential growth curves are shown in S1 Appendix.

The growth rate of *R*. *trifolii* was differentially affected by additions of P25, E171 and NNM TiO_2_ and was significantly reduced by 43% in average (p<0.001) by actual concentration of 23 mg l^-1^ E171 and by 23% (p = 0.035) in 18 mg l^-1^ NNM TiO_2_ treatment (Fig 3, S1 Apendix, and S4 Dataset). The ZnSO_4_*7H_2_O treatment reduced the relative growth rate in average by 90%. Growth curves are shown in S1 Appendix. The lower concentrations of all treatments did not affect the growth rate compared to the control.

### Effects on red clover and *R*. *trifolii*

**Fig 4:**
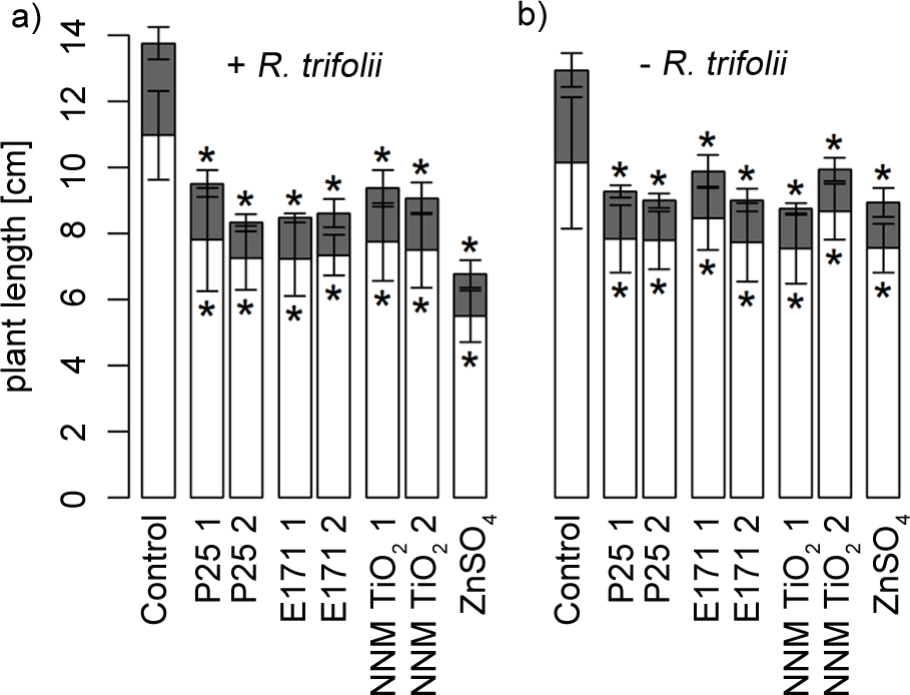
Length of the main root (white) and shoot (grey) at harvest (t = 4 weeks). Lengths are shown for the control, the different TiO_2_ NP treatments in two concentrations 1 (low) and 2 (high) that are described in Table 2, and the 16.1 mg l^-1^ ZnSO_4_ treatment (a) in presence of *R*. *trifolii* or (b) without *R*. *trifolii*. Significant (p<0.05) differences to the respective control (n = 6) are indicated with asterisks above the standard deviation error bars for shoots, and below the error bars for roots. The results of the same treatments with and without *R*. *trifolii* are not significantly different and neither were the controls, but the root length of ZnSO_4_ with and without *R*. *trifolii* was different (p = 0.005).

Shoot and root length of red clover plants significantly (p<0.05) decreased in all three TiO_2_ NP treatments, i.e., P25, E171 and NNM TiO_2_, as well as in the ZnSO_4_ control regardless of the addition of *R*. *trifolii* (Fig 4). Growth reduction ranged between 41 and 62% for shoots and between 26 and 29% for roots, respectively (S1 Appendix and S5 Dataset). Root and shoot dry weight significantly (p<0.05) decreased between 30 and 44% for roots and 27 and 53% for shoots in average over all TiO_2_ NP treatments (S1 Appendix). However, for the two NNM TiO_2_ treatments with *R*. *trifolii*, this reduction of root weight was not significantly different from the control (p = 0.06 and 0.08). Pearson’s correlations between shoot weight and shoot length was moderate with r = 0.67 (p<0.001) and r = 0.62 (p<0.001) with and without *R*. *trifolii* inoculation, respectively. Root morphology, i.e., number of root tips and secondary roots divided by main root length, was not affected by any of the treatments when compared to the controls (Table 3).

**Table 3:** Number of root tips and number of secondary roots of red clover in hydroponic system. Roots were assessed at the harvest (n = 6, mean ± standard deviation). Exposure concentrations (1<2) are described in detail in Table 2.

root architecture	Rhizobia^1^	Control	P25 (1)	P25 (2)	E171 (1)	E171 (2)	NNM TiO_2_ (1)	NNM TiO_2_ (2)	ZnSO_4_
number of root tips per root length	yes	2.2±0.6	2.7±0.9	3.6±0.4	2.5±0.3	2.3±0.7	2.7±0.8	2.6±1.0	2.2±1.0
	no	2.0±1.0	2.4±1.0	2.1±0.4	2.1±0.4	2.5±0.8	3.0±1.2	1.7±0.4	1.9±0.9
number of secondary roots per root length	yes	1.6±0.4	2.1±0.8	1.8±0.4	2.3±0.3	2.0±0.2	2.1±0.5	2.1±0.7	1.7±0.9
	no	1.6±0.5	2.1±0.7	1.7±0.4	1.8±0.2	2.0±0.6	3.0±0.9	1.5±0.3	1.6±0.7

^1^with or without inoculation of *R*. *trifolii*

Red clover formed root nodules in all TiO_2_ NP treatments when *R*. *trifolii* was added. However, the number of nodules decreased significantly (p = 0.02) by 75% compared to the control when treated with ZnSO_4_ (S1 Appendix). No nodules were formed in 2 to 3 of the six replications when treated with E171 (2), NNM TiO_2_ and ZnSO_4_ (S5 Dataset). Plants grown without *R*. *trifolii* also formed nodule-like structures, and at the lower concentration of P25, E171 and NNM TiO_2_ their number increased significantly (p<0.05), by 120%, 80% and 90% compared to the control. Only one control plant without inoculation of *R*. *trifolii* formed nodule-like structures, and none was found in the ZnSO_4_ treatment. To confirm if the nodules were colonized by *R*. *trifolii*, they were plated on YMB agar. Two control nodules and two E171 treated nodules, with inoculation of *R*. *trifolii*, revealed bacterial growth on agar containing rifampicin. This confirms the presence of inoculated rifampicin resistant *R*. *trifolii*. (S6 Dataset). However, nodule-like structures from treatments without inoculation revealed no bacterial growth on YMB agar without rifampicin. Both, 50% of control and E171 treated plants, which were inoculated with *R*. *trifolii*, revealed nodule-like structures which formed no bacterial colonies on the agar plates.

**Fig 5:**
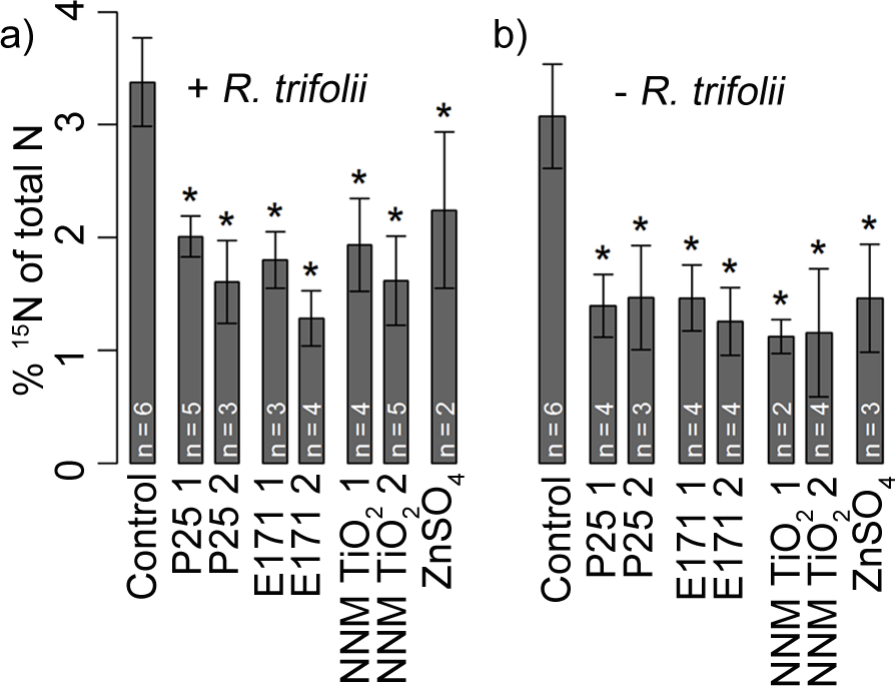
^15^N content (% of total N) of red clover shoots. Results are shown for the control, the different TiO_2_ NPs in two concentrations (1 = low, 2 = high) as described in Table 1 and the ZnSO_4_ treatment (a) with addition of *R*. *trifolii* and (b) without *R*. *trifolii* inoculation. Error bars indicate standard deviations, asterisks show significant differences compared to the control (p<0.05) and number of replications are indicated on the graph (n). Number of replications varied because not for all samples the required amount of shoot biomass for ^15^N measurement was available.

^15^N contents in the shoots decreased in average by 49% in the TiO_2_ NP treated plants with addition of *R*. *trifolii* and 57% without *R*. *trifolii* compared to the control (p <0.001) (Fig 5). Because of too little biomass, not all replications could be assessed for ^15^N content. Shoot content of ^15^N decreased in ZnSO_4_ treated plants with *R*. *trifolii* by 34% and by 52% without *R*. *trifolii* compared to the control (Fig 5). Pearson’s correlation of ^15^N content of shoots and the shoot dry weight was r = 0.61 (p<0.001) for treatments with and r = 0.62 (p<0.001) without inoculation of *R*. *trifolii*. Shoot length was correlated with the ^15^N content in shoots and was r = 0.71 (p<0.001) with inoculation and r = 0.88 (p<0.001) without inoculation of *R*. *trifolii*. The ratio of ^15^N content and biomass was only significantly decreased in the E171 19 mg l^-1^ treatment (S1 Appendix).

## Discussion

### Nanoparticles in growth media

In this study we investigated the potential effects of two different TiO_2_ NPs, i.e., P25, E171, and a non-nanomaterial TiO_2_ referred to as NNM TiO_2_, on *R*. *trifolii* growth in liquid cultures as well as on red clover growth and root nodulation in a hydroponic system. These experiments revealed that exposure concentration changed during the course of the incubation as previously reported for other growth media [19, 40]. It has been reported that growth media for plants and bacteria promote agglomeration and thus sedimentation of TiO_2_ NPs takes place [19, 40]. However, in these studies different media and conditions were used and we aimed at determining the actual Ti-concentrations and NP qualities over time in our system. In the *R*. *trifolii* liquid cultures, the medium was mixed constantly and therefore the concentrations of the suspensions were stable over time. However, in the hydroponic system the medium was not mixed and sedimentation was investigated. We addressed this by periodically changing the medium and determining the actual exposure concentration every week. Even though we applied the same method for the weekly preparations of NP suspensions, it did not always yield the same concentrations. The experimental variation ranged from 11 to 27 mg l^-1^. Even though the exposure concentration was not constant and lower than the nominal concentration, we found effects on red clover plants in all treatments and were able to relate them to actual concentrations. Contrastingly to the primary particle size (P25<E171<NNM TiO_2_) the results showed that P25 formed the largest agglomerates in the growth media while E171 revealed similar agglomerate sizes as NNM TiO_2_ assessed by DLS (Tables 1 and 2). P25 sedimented faster than E171 in the hydroponic system (Fig 1 and S1 Appendix). However, actual particle size and actual NP concentration of the suspensions were not correlated (p = 0.647). Sedimentation of the NPs is also depended on the zeta potential [41]. The measured zeta potentials were moderately correlated with the actual Ti-concentration (r = -0.48, p = 0.013) and with the measured NP sizes (r = 0.55, p<0.001) but did not explain the whole variation. P25 revealed a less negative zeta potential than E171 and NNM TiO_2_ confirming the finding that P25 sedimented faster than E171. It is not known how stable TiO_2_ agglomerates are in these systems and which proportion of free NPs are occurring. These free NPs potentially have stronger effects on plants and microbes than the agglomerates.

### Effects of TiO_2_ NPs on *R*. *trifolii* in liquid cultures

We first tested effects of TiO_2_ NPs on the growth of *R*. *trifolii* in liquid cultures before we went to the more complex system with plants and bacteria. In liquid cultures with P25 (up to 23 mg l^-1^, 806±17 nm, mixture of anatase and rutile) *R*. *trifolii* growth rate was not affected. The two anatase preparations E171 and NNM TiO_2_ with average agglomerate sizes of 341±3 nm and 356±1 nm, respectively, decreased the relative growth rate. E171 and NNM TiO_2_ had different primary particle sizes, i.e., 92±31 and 145±46 nm, but did not reveal differences in affecting *R*. *trifolii* growth. This is in agreement with the similar agglomerate size of E171 and NNM TiO_2_. The increase of OD of the medium containing P25 and controls was not different and therefore was indicative for bacterial growth. Different photocatalytic activities of the anatase particles and the mixture of anatase and rutile might affect plants and bacteria. However, our experiments were conducted under dark conditions and thus effects of reactive oxygen species were excluded. [42] used another bacterial model species and reported that 25 mg l^-1^ anatase affected the viability of *Escherichia coli* stronger than the same concentration of a mixture of 93% anatase and 7% rutile. The viability has been reduced by 40 and 25%, respectively. However, in the study of Lin et al.[42], the experiments were performed under natural light conditions and reactive oxygen species were released resulting in stronger effects of smaller anatase particles than larger particles or particles with a rutile crystal structure.

### Effects of TiO_2_ NPs on red clover and the formation of root nodules in a hydroponic system

Based on the information that E171 and NNM TiO_2_ affected bacterial growth in liquid cultures, we performed experiments in a more complex hydroponic systems using red clover and *R*. *trifolii*. The shoot as well as root length and weight decreased significantly in TiO_2_ NP treatments and the ZnSO_4_ control. For the ZnSO_4_ treated plants, this growth reduction was similar to other studies with comparable Zn^2+^ concentrations [28–29]. Different plant species might be affected differently by TiO_2_ NPs. In contrast to our investigations of decreased shoot length in TiO_2_ NP treatments, no effects on shoot length have been reported for pea when exposed to 250 mg P25 l^-1^ [15] and for cucumber even an increase in shoot length at 4000 mg TiO_2_ NPs l^-1^ has been observed [43]. While shoot length, root length and shoot weight decreased in all treatments in our experiments, root dry weight was not affected by NNM TiO_2_ because there was a higher variance between replications. This might be because of the larger primary particle size of NNM TiO_2_ compared to E171 and P25, which both decreased root weight. However, the mechanism how these NPs affect root weight is not known. In our experiment with red clover, we did not find effects on the number of secondary roots or the number of root tips as reported for pea [15].

In the hydroponic system it was not possible to determine nitrogen fixation because plants from the same treatment with and without nodules revealed the same ^15^N signature. The number of plants, which did not form nodules when inoculated with *R*. *trifolii*, were increased in TiO_2_ NP treatments, which might result from decreased bacterial growth observed in the liquid cultures. For another legume-rhizobium system (pea and *R*. *leguminosarum bv*. *viciae*) Fan et al. [15] have reported that nodule formation was delayed. Nodulation could also be influenced by adhesion of TiO_2_ NPs on root hairs, or TiO_2_ NPs might interact with the signaling compounds (flavonoids, lipo-oligosaccharides). Further research is needed to understand the mechanism how TiO_2_ NPs affect nodulation because reduction of root nodules would influence nitrogen fixation and thereby an important ecosystem function. Nodules, which revealed bacterial growth on YMB agar, grew also on agar containing rifampicin. This implies that these nodules were colonized by the inoculated rifampicin resistant *R*. *trifolii* and it was independent of the NP treatment. Interestingly, we found also nodule-like structures in the TiO_2_ NP treatments without inoculation of *R*. *trifolii*. None of the tested nodule-like structures did reveal bacterial growth on YMB agar independent if they originated form E171 treated plants or controls. This implies, that these nodules were either not colonized or responded differently to the surface sterilization than the nodules, which revealed bacterial growth on YMB agar with rifampicin. It has been reported, that low concentrations of nitrogen in a medium can enhance the spontaneous production of nodule-like structures in white clover [44]. Red clover plants treated with TiO_2_ NPs and ZnO_4_ in our experiment revealed a reduced ^15^N content in shoots. Therefore the limitation of nitrogen in red clover might have induced these nodule-like structures in our experiment. However, more research is needed to understand the mechanism. We could use the ^15^N content of shoots as a proxy of nutrient uptake. All treatments revealed a decreased ^15^N content in shoots, which indicated a reduced nutrient uptake compared to the controls. However, due to insufficient quantities of biomass not all replications could be used for ^15^N analysis. Therefore some of the treatments did not have enough replicates for giving enough statistical power. Nevertheless, the results clearly indicate a reduced nitrogen uptake of the red clover plants. For peas Fan et al. [15] have reported that plants treated with TiO_2_ NPs revealed impaired water uptake and Asli and Neumann [45] have reported reduced transpiration in corn treated with 1 g l^-1^ P25. Adhesion of NPs on root surfaces has been discussed as possible mechanism [15, 45]. Pores in the cell walls of plants are approximately 5 nm in diameter [46] and thus might be blocked by the TiO_2_ NPs and agglomerates. To investigate this further and to test whether the TiO_2_ NP covered the roots, we performed scanning electron microscopy to assess TiO_2_ NPs on the root surface. Only 1% of the root surface was covered by E171 agglomerates or single particles, indicating sparse or loose attachment of TiO_2_ NPs to the roots. Investigation of uptake of TiO_2_ NP into red clover plants was not the aim of this study which would have required larger plants yielding more biomass.

## Conclusions

TiO_2_ NPs agglomerated and revealed particle sizes larger than 100 nm in growth media. They tended to sediment in the hydroponic system and thereby decreasing the actual exposure concentrations, demonstrating the importance of determining the actual exposure concentration. Anatase TiO_2_ NPs, i.e., E171 and NNM TiO_2_, significantly reduced growth rate of *R*. *trifolii* and did not display a primary particle size dependent effect because they reduced bacterial growth in the same extent and revealed similar aggregate sizes. In the hydroponic system, red clover biomass significantly decreased in all TiO_2_ NP treatments and NNM TiO_2_. Red clover plants treated with TiO_2_ NPs revealed reduced nitrogen (^15^N) content, indicating impaired nutrition and elevated stress. P25, E171 and NNM TiO_2_ did affect red clover and *R*. *trifolii* in artificial hydroponic cultures, but it remains to be tested which mechanisms are responsible for these effects and whether these effects extend to plants grown in soil.

## Supporting Information

S1 Appendix Supplementary Information. Method for scanning electron microscopy, transmission electron microscopy, transmission electron microscopy of primary particles, *R*. *trifolii* growth curves, picture of the hydroponic system, and statistical outputs of the experiments.
(DOCX)

S1 Dataset Titanium concentration in YMB. (TXT)

S2 Dataset NP size and zeta potential of the hydroponic experiment. (TXT)

S3 Dataset Measured titanium concentration in the sedimentation experiment. (TXT)

S4 Dataset Optical density data of *R*. *trifolii* liquid growth in cultures. (TXT)

S5 Dataset Measured endpoints of the hydroponic experiment. (TXT)

S6 Dataset Bacterial colony forming units from nodules plated on agar. (TXT)

S1 Fig Transmission electron microscopy pictures of nanoparticles. From the left to the right: non-nanomaterial (NNM) TiO_2_ particles, E171 and P25 nanoparticles.
(TIF)

S2 Fig *Rhizobium trifolii* growth curves. Measured by optical density (OD) at 620 nm over time for a) P25, b) E171 and c) NNM TiO_2_. Increasing concentrations of TiO_2_ NPs are indicated in red, green, blue and cyan for 1, 3, 8 and 23 mg l^-1^ for P25 and E171 and 1, 2, 6 and 18 mg l^-1^ for NNM TiO_2_. Each of the three experiments contained a control (black circles) and a positive control (gray circles), i.e. ZnSO_4_*7H_2_O at 12.5 mg l^-1^. Four replications of each treatment are shown. To remove the NP background of OD, we measured the same concentrations of NPs in YMB without *R*. *trifolii* and subtracted this value from the samples with *R*. *trifolii*.
(TIF)

S3 Fig Hydroponic system after three weeks of growth. Shown are two replications of the control as well as the E171 1 and E171 2 treatments. On the drawing the setup of the hydroponic system is explained.
(TIF)
