# Supplementary material for: Effects of Titanium Dioxide Nanoparticles on Red Clover and Its Rhizobial Symbiont
Source: PLoS One. 2016 May 12;11(5):e0155111. doi: 10.1371/journal.pone.0155111 (PMC4865228; doi:10.1371/journal.pone.0155111)
Supplement: S1 Appendix — Method for scanning electron microscopy, transmission electron microscopy, transmission electron microscopy of primary particles, R. trifolii growth curves, picture of the hydroponic system, and statistical outputs of the experiments. (DOCX) [file pone.0155111.s001.docx]

# Supplementary information

# Effects of Titanium Dioxide Nanoparticles on Red Clover and its Rhizobial Symbiont

Janine Moll^1^, Annette Okupnik^1,#a^, Alexander Gogos^1,#b^, Katja Knauer^2^, Thomas D. Bucheli^1^, Marcel GA van der Heijden^1^, Franco Widmer^1^*

^1^Institute for Sustainability Sciences ISS, Agroscope, Zurich, Switzerland
^2^Federal Office for Agriculture FOAG, Berne, Switzerland

## S1 Text: Scanning electron microscopy of red clover roots.

Root samples of a repetition of the hydroponic experiment with 24 mg E171 l^-1^ were analyzed by scanning electron microscopy for coverage of titanium particles (Figure 2). Dried red clover roots were fixed on a conductive adhesive tape, mounted on an aluminum sample holder and sputtered with a 3 nm layer of platinum using a Bal-tec MED020 sputter coater (Bal-Tec, Liechtenstein). Samples were imaged using a Zeiss Supra 50VP scanning electron microscope (SEM) operating at 10kV. An energy dispersive x-ray (EDX) spectrometer (x-act, Oxford Instruments, United Kingdom) was employed for elemental analysis of selected areas on the root surface. The coverage of the TiO_2_ NPs on the root surface was assessed.


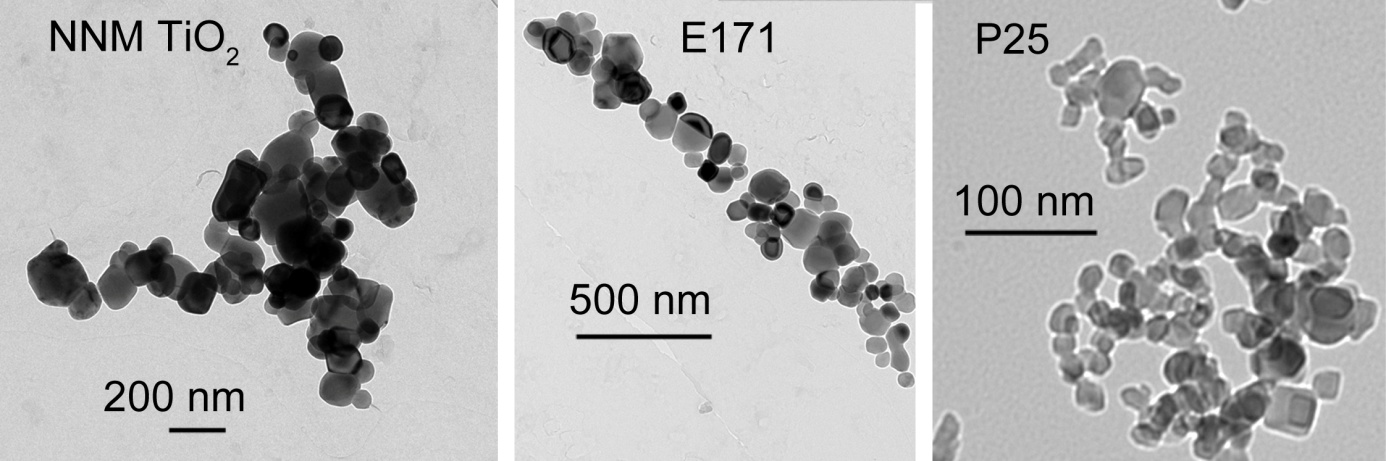


**S1 Fig. Transmission electron microscopy pictures of nanoparticles.** From the left to the right: non-nanomaterial (NNM) TiO_2_ particles, E171 and P25 nanoparticles.

**
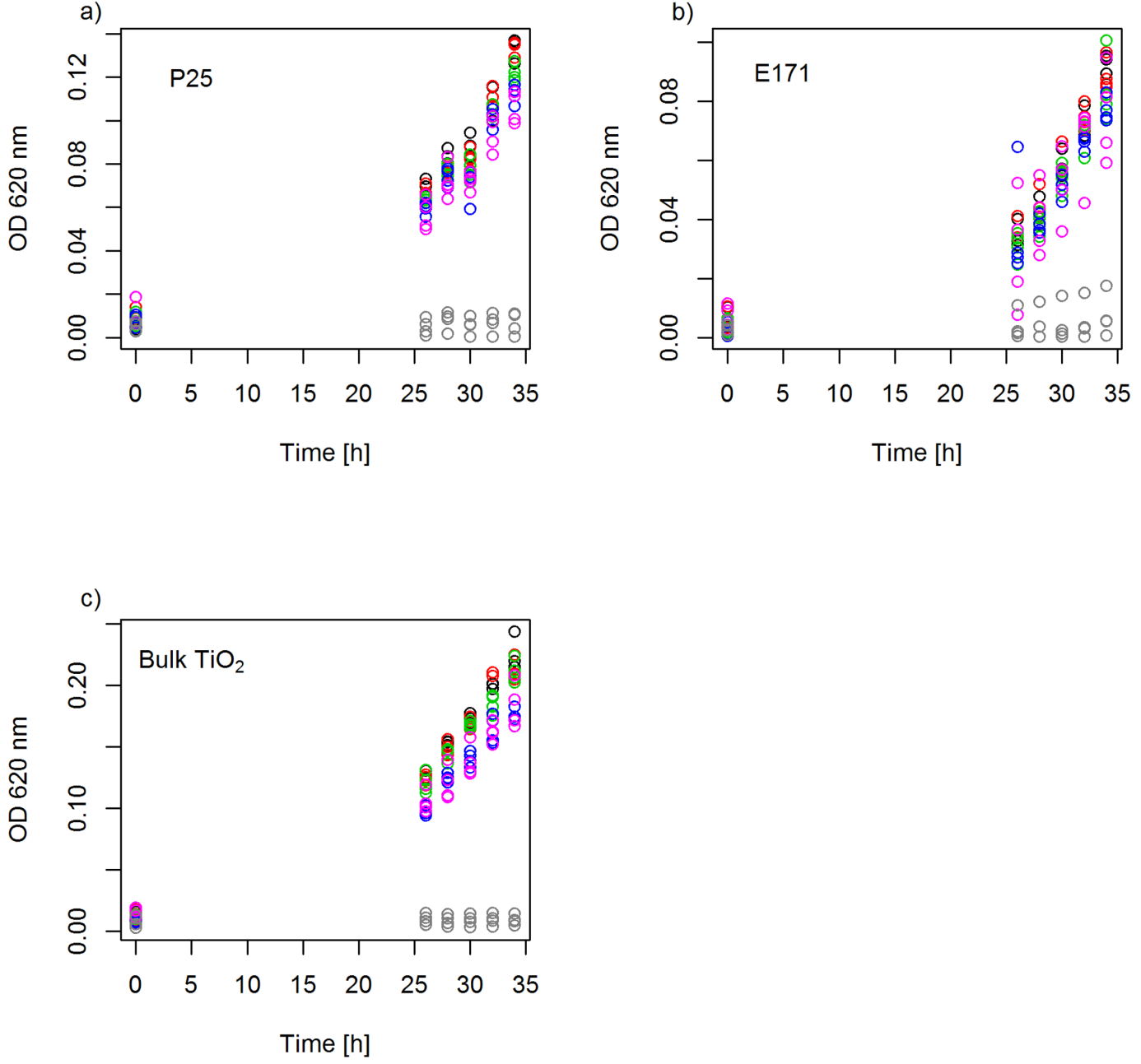
**

**S2 Fig. *Rhizobium trifolii* growth curves.** Measured by optical density (OD) at 620 nm over time for a) P25, b) E171 and c) NNM TiO_2_. Increasing concentrations of TiO_2_ NPs are indicated in red, green, blue and cyan for 1, 3, 8 and 23 mg l^-1^ for P25 and E171 and 1, 2, 6 and 18 mg l^-1^ for NNM TiO_2_. Each of the three experiments contained a control (black circles) and a positive control (gray circles), i.e. ZnSO_4_*7H_2_O at 12.5 mg l^-1^. Four replications of each treatment are shown. To remove the NP background of OD, we measured the same concentrations of NPs in YMB without *R. trifolii* and subtracted this value from the samples with *R. trifolii*.

**
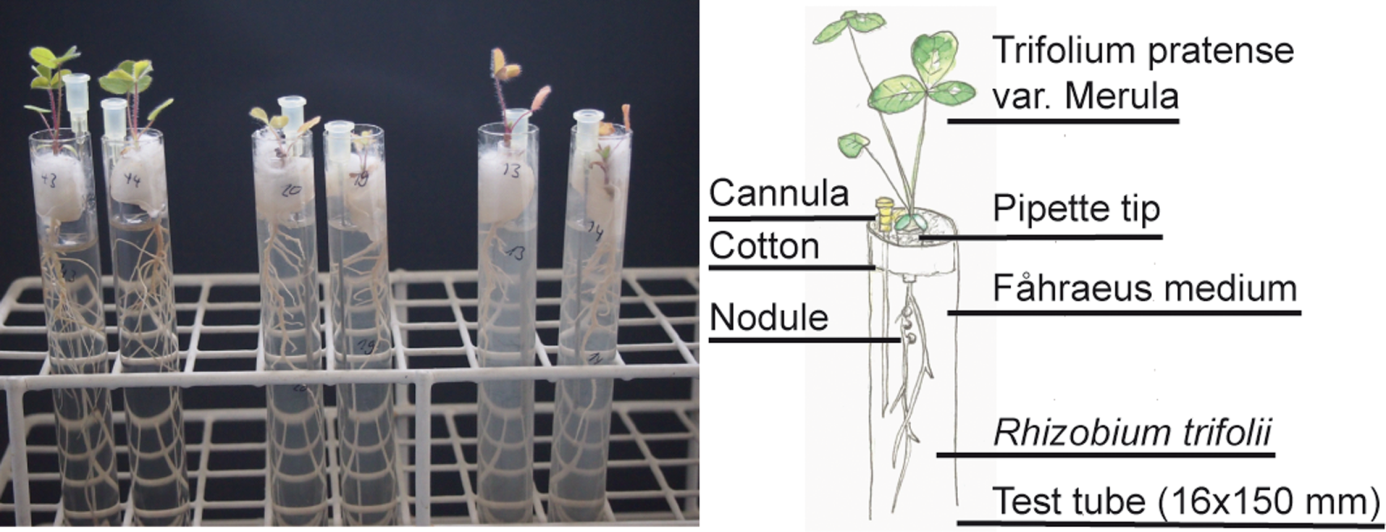
**

**S3 Fig. Hydroponic system after three weeks of growth.** Shown are two replications of the control as well as the E171 1 and E171 2 treatments. On the drawing the setup of the hydroponic system is explained.

**S1** **Table.Statistics for titanium concentration over time.** Generalized linear model of total elemental titanium concentration over time in the Fåhraeus medium. Statistics are shown for the hydroponic system for the two particles E171 and P25 compared to the initial concentration. P-values were adjusted for multiple testing by Beniamini-Hochberg adjustment using the R command p.adjust.

| **glm** | **E171** | |  | **P25** | |
| --- | --- | --- | --- | --- | --- |
| **time [h]** | **t** | **p_adj_** |  | **t** | **p_adj_** |
| 18 | -0.6 | 0.591 |  | -1.3 | 0.215 |
| 24 | -1.3 | 0.276 |  | -2.5 | **0.034** |
| 42 | -3.6 | **0.005** |  | -2.9 | **0.020** |
| 114 | -7.1 | **<0.001** |  | -3.8 | **0.006** |
| 162 | -7.0 | **<0.001** |  | -3.7 | **0.006** |

**S2 Table. Statistics for *R. trifolii* growth rates.** Generalized linear model (glm) of the *R. trifolii* growth rates (slope of exponential growth phase) compared to the control. Statistics is shown for the different concentrations and NPs, i.e., P25, E171 and NNM TiO_2_. Concentrations for P25 and E171 are shown and the ones for NNM TiO_2_ are in parentheses. P-values were adjusted according to Benjamini-Hochberg using the R command p.adjust. In the case where the model assumptions were not fulfilled, a Kruskal-Wallis test was performed additionally.

| **glm** | **P25** | |  | **E171^a^** | | |  | **NNM-TiO2** | |
| --- | --- | --- | --- | --- | --- | --- | --- | --- | --- |
| **concentration [mg l^-1^]** | **t** | **p_adj_** |  | **t** | **p_adj_** | **Mann-Whitney p** |  | **t** | **p_adj_** |
| 1 (1) | 1.8 | 0.181 |  | -0.7 | 0.659 | 0.200 |  | -0.5 | 0.758 |
| 3 (2) | 0.3 | 0.867 |  | 0.0 | 0.971 | 0.886 |  | -0.3 | 0.758 |
| 8 (6) | -0.2 | 0.867 |  | -1.3 | 0.408 | 0.200 |  | -0.4 | 0.758 |
| 23 (18) | 2.3 | 0.132 |  | -3.0 | **0.034** | **0.029** |  | -5.0 | **0.001** |

^a^Resudials were not normally distributed, therefore an additiona Kruskal-Wallis test
Test was applied revealing a significant difference (p=0.029).

**S3 Table. Statistics of the hydroponic experiment with inoculation of *R. trifolii*.** Results of the statistical tests from the hydroponic experiment with *R. trifolii* inoculation. For each variable a generalized linear model (glm) was performed. In the case where the residuals were not normal distributed, a Mann-Whitney test was applied. P-values are adjusted for multiple testing. Data are shown in S1 Dataset. Exposure concentrations 1 and 2 (1=low, 2=high) are described in detail in Table 2.

|  | **root length** | | **shoot length** | | **root weight** | | **shoot weight** | | **number of nodules** | | **number of root tips per root lenght** | | **secundary roots per length** | | **^15^N content shoot** | | **^15^N concent soot / plant weight** | |
| --- | --- | --- | --- | --- | --- | --- | --- | --- | --- | --- | --- | --- | --- | --- | --- | --- | --- | --- |
|  | **glm t-value** | **glm p-value** | **glm t-value** | **glm p-value** | **Mann -Whitney W** | **Mann-Whitney p-value** | **glm t-value** | **glm p-value** | **glm z-value** | **glm p-value** | **glm t-value** | **glm p-value** | **glm t-value** | **glm p-value** | **glm t-value** | **glm p-value** | **glm t-value** | **glm p-value** |
| ***R. trifolii* inoculation** | **yes** | **yes** | **yes** | **yes** | **yes** | **yes** | **yes** | **yes** | **yes** | **yes** | **yes** | **yes** | **yes** | **yes** | **yes** | **yes** | **yes** | **yes** |
| P25 9.0 mg l^-1^ | -4.9 | **<0.001** | -4.5 | **<0.001** | 33 | **0.039** | -4.6 | **<0.001** | 0.9 | 0.432 | 1.0 | 0.688 | 1.4 | 0.307 | -5.7 | **<0.001** | 0.2 | 0.822 |
| P25 18.0 mg l^-1^ | -5.7 | **<0.001** | -7.1 | **<0.001** | 34 | **0.027** | -4.6 | **<0.001** | -1.2 | 0.302 | 1.9 | 0.249 | 1.2 | 0.307 | -7.3 | **<0.001** | -0.6 | 0.712 |
| E171 9.5 mg l^-1^ | -5.8 | **<0.001** | -6.3 | **<0.001** | 34 | **0.027** | -5.2 | **<0.001** | 2.6 | **0.001** | 0.6 | 0.763 | 2.0 | 0.211 | -6.2 | **<0.001** | -0.2 | 0.822 |
| E171 19.0 mg l^-1^ | -5.6 | **<0.001** | -6.3 | **<0.001** | 33 | **0.039** | -4.7 | **<0.001** | -0.7 | 0.506 | 0.2 | 0.908 | 1.0 | 0.356 | -9.0 | **<0.001** | -1.6 | 0.300 |
| NNM TiO_2_ 9.5 mg l^-1^ | -5.0 | **<0.001** | -4.8 | **<0.001** | 31 | 0.062 | -3.2 | **0.002** | -1.6 | 0.178 | 1.1 | 0.687 | 1.2 | 0.307 | -6.2 | **<0.001** | -1.2 | 0.468 |
| NNM TiO_2_ 19.0 mg l^-1^ | -5.4 | **<0.001** | -5.1 | **<0.001** | 31 | 0.055 | -3.8 | **<0.001** | -2.1 | 0.057 | 0.8 | 0.688 | 1.3 | 0.307 | -8.1 | **<0.001** | -1.8 | 0.300 |
| ZnSO_4_ 16 mg l^-1^ | -8.5 | **<0.001** | -6.2 | **<0.001** | 36 | **0.017** | -5.0 | **<0.001** | -2.8 | **0.015** | -0.1 | 0.908 | 0.3 | 0.762 | -3.9 | **<0.001** | 1.1 | 0.468 |

**S4 Table.** **Statistics of the hydroponic experiment with inoculation of *R. trifolii*.** Results of the statistical tests from the hydroponic experiment without *R. trifolii* inoculation. For each variable a generalized linear model (glm) was performed. In the case where the residuals were not normal distributed, a Mann-Whitney test was applied. P-values are adjusted for multiple testing. Data are shown in S1 Dataset. Exposure concentrations 1 and 2 (1=low, 2=high) are described in detail in Table 2.

|  | **root length** | | **shoot length** | | **root weight** | | **shoot weight** | | **number of nodules** | | **number of root tips per root lenght** | | **secundary roots per length** | | **^15^N content shoot** | | **^15^N concent soot / plant weight** | |
| --- | --- | --- | --- | --- | --- | --- | --- | --- | --- | --- | --- | --- | --- | --- | --- | --- | --- | --- |
| **without *R. trifolii* inoculation** | **glm t-value** | **glm p-value** | **glm t-value** | **glm p-value** | **glm t-value** | **glm p-value** | **Mann -Whitney W** | **Mann-Whitney p-value** | **glm z-value** | **glm p-value** | **glm t-value** | **glm p-value** | **glm t-value** | **glm p-value** | **glm t-value** | **glm p-value** | **glm t-value** | **glm p-value** |
| P25 9.0 mg l^-1^ | -3.5 | **0.002** | -6.5 | **<0.001** | -2.8 | **0.009** | 34 | **0.008** | 3.4 | **0.005** | 0.9 | 0.726 | 1.5 | 0.363 | -6.4 | **<0.001** | -1.6 | 0.157 |
| P25 18.0 mg l^-1^ | -3.6 | **0.002** | -7.7 | **<0.001** | -3.1 | **0.006** | 32 | **0.030** | 1.1 | 0.438 | 0.2 | 0.867 | 0.3 | 0.907 | -5.6 | **<0.001** | -2.0 | 0.115 |
| E171 9.5 mg l^-1^ | -2.5 | **0.017** | -6.6 | **<0.001** | -2.6 | **0.015** | 32 | **0.026** | 2.9 | **0.009** | 0.2 | 0.867 | 0.4 | 0.907 | -6.1 | **<0.001** | -1.9 | 0.115 |
| E171 19.0 mg l^-1^ | -3.5 | **0.002** | -6.9 | **<0.001** | -2.3 | **0.025** | 27 | **0.040** | 0.2 | 0.978 | 1.0 | 0.726 | 1.2 | 0.439 | -6.9 | **<0.001** | -2.8 | **0.041** |
| NNM TiO_2_ 9.5 mg l^-1^ | -3.9 | **<0.001** | -7.7 | **<0.001** | -3.3 | **0.005** | 30 | 0.065 | 3.1 | **0.008** | 1.4 | 0.686 | 2.0 | 0.218 | -5.8 | **<0.001** | -1.5 | 0.160 |
| NNM TiO_2_ 19.0 mg l^-1^ | -2.2 | **0.031** | -7.3 | **<0.001** | -3.1 | **0.006** | 32 | **0.026** | 0.8 | 0.565 | -0.6 | 0.867 | -0.4 | 0.907 | -7.2 | **<0.001** | -2.0 | 0.115 |
| ZnSO_4_ 16 mg l^-1^ | -3.0 | **<0.001** | -6.8 | **<0.001** | -5.3 | **<0.001** | 35 | **0.004** | 0.0 | 0.991 | -0.2 | 0.867 | 0.0 | 0.98 | -5.6 | **<0.001** | 0.5 | 0.601 |

**S1 Dataset. Measured endpoints of the hydroponic experiment.**

**S2 Dataset. NP size and zeta potential of the hydroponic experiment.**

**S3 Dataset. Measured titanium concentration in the sedimentation experiment.**

**S4 Dataset. Optical density data of *R. trifolii* liquid growth in cultures.**

**S5 Dataset. Titanium concentration in YMB.**

**S6 Dataset. Bacterial colony forming units from nodules plated on agar.**
