# Supplementary figures and images for: Effects of Titanium Dioxide Nanoparticles on Red Clover and Its Rhizobial Symbiont
Source: PLoS One. 2016 May 12;11(5):e0155111. doi: 10.1371/journal.pone.0155111 (PMC4865228; doi:10.1371/journal.pone.0155111)

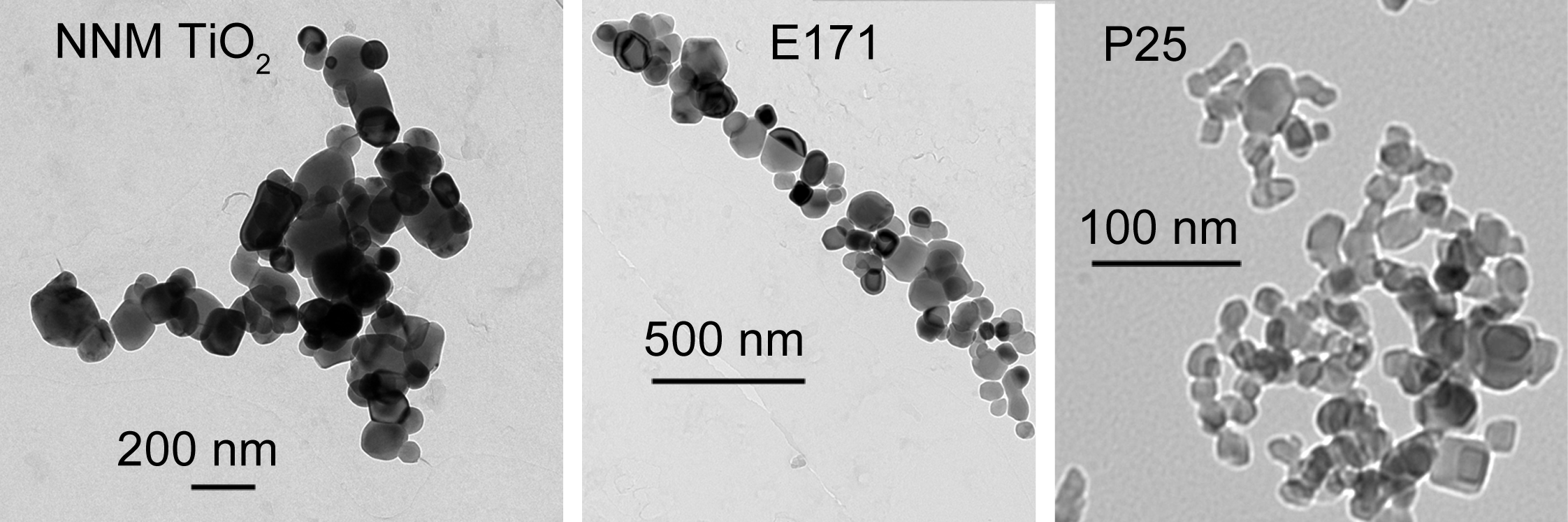

Supplement: S1 Fig — From the left to the right: non-nanomaterial (NNM) TiO2 particles, E171 and P25 nanoparticles. (TIF) [file pone.0155111.s008.tif]

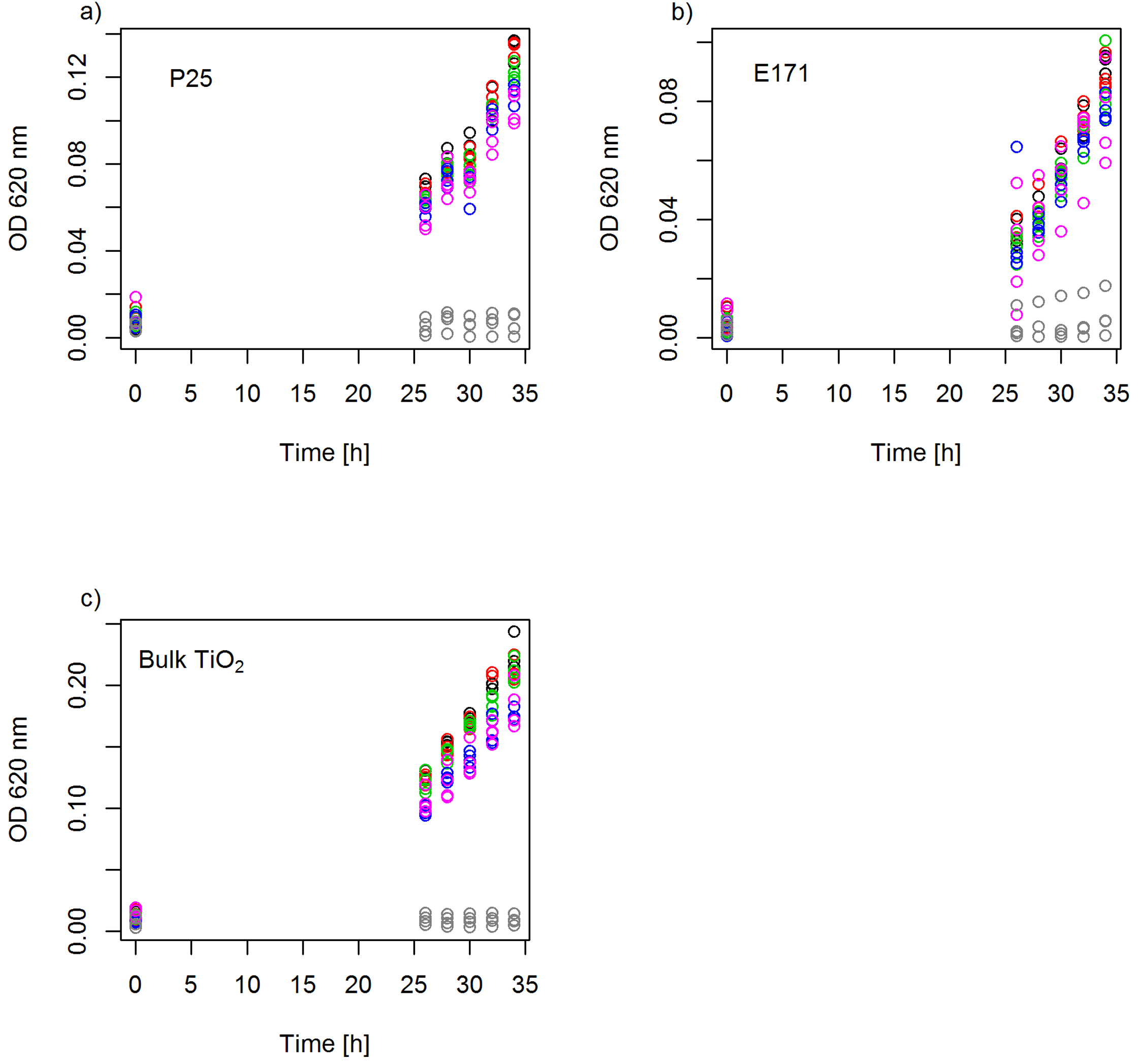

Supplement: S2 Fig — Measured by optical density (OD) at 620 nm over time for a) P25, b) E171 and c) NNM TiO2. Increasing concentrations of TiO2 NPs are indicated in red, green, blue and cyan for 1, 3, 8 and 23 mg l-1 for P25 and E171 and 1, 2, 6 and 18 mg l-1 for NNM TiO2. Each of the three experiments contained a control (black circles) and a positive control (gray circles), i.e. ZnSO4*7H2O at 12.5 mg l-1. Four replications of each treatment are shown. To remove the NP background of OD, we measured the same concentrations of NPs in YMB without R. trifolii and subtracted this value from the samples with R. trifolii. (TIF) [file pone.0155111.s009.tif]

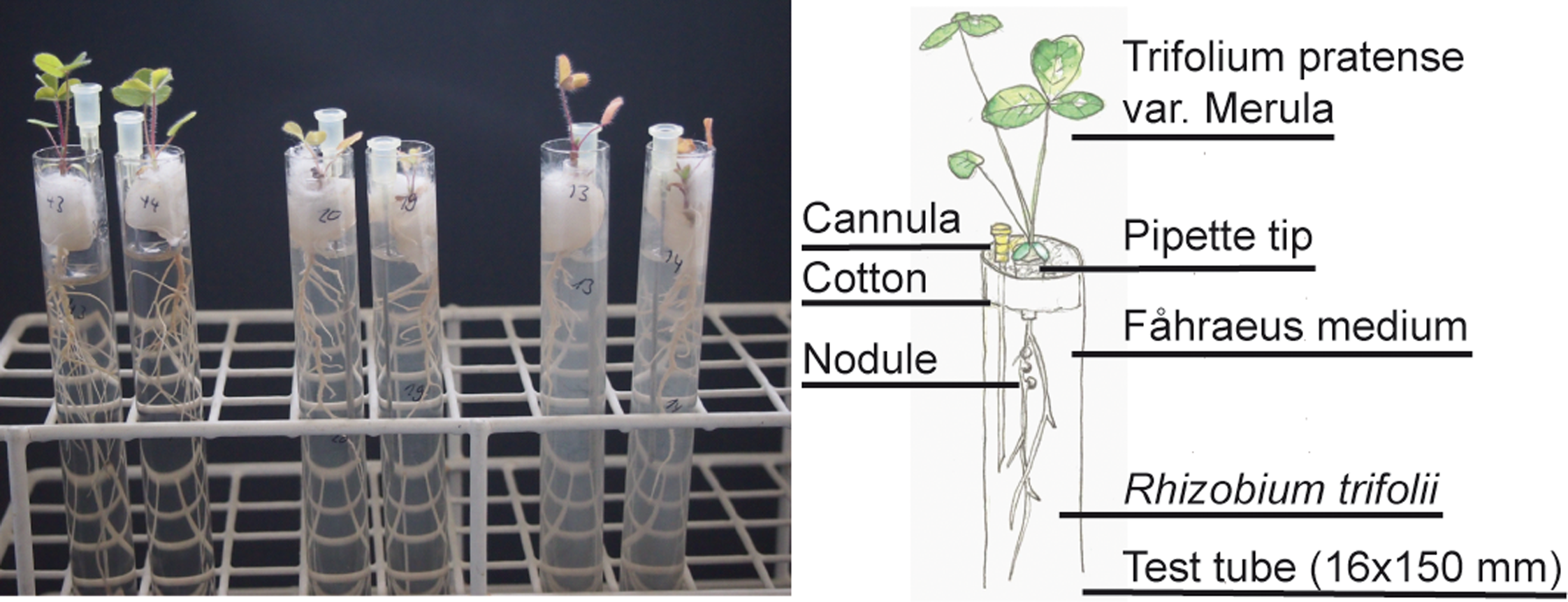

Supplement: S3 Fig — Shown are two replications of the control as well as the E171 1 and E171 2 treatments. On the drawing the setup of the hydroponic system is explained. (TIF) [file pone.0155111.s010.tif]
